# Factors Affecting Follow-up for Specialty Postpartum Care after Obstetric Anal Sphincter Injury at a Single U.S. Institution

**DOI:** 10.1007/s00192-025-06126-w

**Published:** 2025-04-15

**Authors:** Simone Reaves, Pamela J. Levin, Heidi S. Harvie, Uduak U. Andy

**Affiliations:** Division of Urogynecology and Pelvic Reconstructive Surgery, Department of Obstetrics and Gynecology, University of Pennsylvania, Philadelphia, PA USA

**Keywords:** Depression, Health disparities, Obstetric anal sphincter injury, Postpartum

## Abstract

**Introduction and Hypothesis:**

Early postpartum specialist care may improve outcomes for birthing people who sustain obstetric anal sphincter injuries (OASIS). This study was aimed at describing follow-up rates in a postpartum recovery clinic (PPRC) for patients who sustained OASIS at delivery, and at identifying factors associated with failure to follow up. We hypothesized that providing care in PPRC would result in high rates of access to specialized care for OASIS.

**Methods:**

This was a retrospective cohort study of patients with OASIS at a single institution from January 2018 to December 2023. Patients who sustain OASIS receive an automatic referral to PPRC within 3 weeks postpartum. Demographic and follow-up data were extracted from the medical records, including Edinburgh Postpartum Depression Scale (EPDS) score. The primary outcome was follow-up in PPRC. We examined associations between patient characteristics and failure to follow up using univariable and multivariable logistic regression.

**Results:**

Among 659 deliveries with OASIS during the study period, 540 (81.9%) followed up in a PPRC and of those 468 (86.7%) followed up within 3 weeks. Failure to follow up was associated with multiparity, Black race, Hispanic ethnicity, having Medicaid or state insurance, and elevated EPDS score on univariable analysis. On multivariable analysis, having Medicaid or state medical assistance and elevated depression screening remained associated with failure to follow up in a PPRC.

**Conclusions:**

There was a high overall attendance rate at a urogynecologist-led postpartum clinic in birthing people who sustained OASIS. An elevated depression screening score and having Medicaid or state medical assistance were associated with failure to follow up.

## Introduction

Obstetric anal sphincter injuries (OASIS) during childbirth are relatively uncommon, with an estimated incidence of less than 5% of all vaginal deliveries [1]. However, injury to the anal sphincter carries both short-term and long-term morbidity. Birthing people who sustain OASIS are at a higher risk for complications such as perineal wound infection and breakdown [2, 3], anal incontinence [4–6], perineal pain, and dyspareunia [7, 8].

Early postpartum evaluation, especially by a specialist, has the potential to improve outcomes for women with postpartum pelvic-floor disorders (PFDs) [9]. Therefore, many institutions are now creating clinics specifically for postpartum perineal care [9–11]. The American College of Obstetricians and Gynecologists (ACOG) recommends that all birthing people have a postpartum visit within 3 weeks of giving birth [12]. However, historically, rates of attendance to postpartum visits has been low. About 40% of people never attend a postpartum visit and people from low-resource settings are less likely to attend a postpartum visit [12].

In addition to these disparities in postpartum care, disparities also exist in PFD care [13]. Specialized postpartum pelvic-floor clinics such as our institution’s urogynecologist-led Postpartum Recovery Clinic (PPRC) are uniquely poised to provide early access to subspecialized care after OASIS for minoritized populations [13]. However, there is a gap in the literature examining if utilization of this care model achieves equity in postpartum care.

This study was aimed at describing follow-up rates in PPRC for patients who had OASIS at delivery, and at identifying factors associated with failure to follow up. We hypothesized that providing care in PPRC would result in high rates of access to specialized care for OASIS for birthing people from diverse backgrounds.

## Materials and Methods

We conducted a retrospective cohort study using the electronic medical records (EMRs) at a single institution in the USA from January 2018 to December 2023. This study met the criteria for IRB review exemption authorized by 45 CFR 46.104, category 2. Patients who sustained third- and fourth-degree perineal lacerations during a vaginal delivery were identified based on Delivery Report data during the study period. Demographic data were extracted from the medical records, including age, self-identified race and ethnicity, primary language spoken at home, parity, and insurance type. We extracted the Edinburgh Postpartum Depression Scale (EPDS) score, which is routinely assessed in all birthing people prior to hospital discharge. The EPDS is a widely used validated screening tool for postpartum depression [14]. It is a self-reported ten-item scale, with scores that range from 0 to 30, with a score ≥ 10 indicating an increased risk for depression [15]. Obstetric data extracted from the delivery summary or delivery note included the gestational age at delivery, delivery method, performance of an episiotomy, birthweight, and the presence of an OASI. OASIS are classified using standard definitions based on the amount of anal sphincter injury [16]. Third-degree lacerations indicate injury to the anal sphincter complex and are subclassified into 3a (a tear of less than 50% of the external anal sphincter [EAS]), 3b (a tear of more than 50% of the EAS), and 3c (a tear of the EAS and the internal anal sphincter [IAS]). A fourth-degree perineal laceration is a laceration involving both the EAS and IAS as well as the anal epithelium.

At our institution, in addition to their routine postpartum visit with their obstetric provider, patients who sustain OASIS receive an automatic referral to the PPRC within 3 weeks postpartum. During the PPRC visit, patients are seen by a urogynecologist and are asked about existing urinary symptoms and bowel symptoms. A pelvic examination is performed to examine the perineal wound for signs of infection or dehiscence, but the examination is limited to the distal vagina for patient comfort. After the initial visit, patients are typically seen for follow-up visits every 2–3 weeks during the healing process. Interventions during this time period may include treatment of wound infection with oral antibiotics and treatment of granulation tissue with silver nitrate. Once the perineal wound has completely healed, patients are referred to pelvic floor physical therapy. PPRC patients are also counseled regarding their injury and offered anorectal testing (endoanal ultrasound and anorectal manometry) to evaluate anal-sphincter integrity and function. These tests can be done at any point after 12 weeks postpartum. Patients who attend the PPRC are also referred to other specialists as needed, such as psychologists for depressed mood or colorectal surgeons for anal fissures or hemorrhoids. Our PPRC accepts private insurance as well as Medicaid. Attendance at the PPRC was assessed from the visit record in the EMR and time to follow-up was assessed as the number of days between the hospital discharge and the PPRC clinic visit. For this analysis, patients were categorized as attending PPRC if they were seen within 180 days of hospital discharge.

Our primary outcome of interest was follow-up in the PPRC, and the secondary outcome was follow-up within 3 weeks of hospital discharge. Descriptive statistics were used to describe the characteristics of the study population. Logistic regression models were used to estimate univariable and multivariable-adjusted odds ratios (ORs) and their 95% confidence intervals (CIs) for follow-up in the PPRC according to various demographic and patient characteristics, including race, ethnicity, primary language, insurance status, and EPDS score. In secondary analyses, we examined the associations between the various demographic and patient characteristics and the secondary outcome of follow-up within 3 weeks. Unadjusted models are compared with models adjusted for all the significant characteristics. Individuals with missing data were considered missing completely at random and were not included in the analysis. STATA version 18 (Stata Corp., College Station, TX, USA) was used for analysis.

## Results

There were 659 deliveries with OASIS during the study period. Demographic and obstetric characteristics of the study cohort are listed in Table 1. The study population was majority white (431 out of 659, 65.4%), non-Hispanic (605 out of 655, 92.4%), and had private insurance (557 out of 659, 84.5%). The mean age at the time of delivery was 31.7 years. Most patients (71.5%) had a spontaneous vaginal delivery, and the majority of patients in the cohort had a third-degree perineal laceration, with 3a laceration being the most common laceration type overall (50.2%). Only 50 patients (7.6%) in the study cohort had a fourth-degree laceration.

**Table 1:** Patient characteristics and attendance to postpartum recovery clinic (PPRC), 2018–2023

	Total cohort (*n* = 659)	Did not attend PPRC (*n* = 119)	Attended PPRC (*n* = 540)	*p* value
Maternal age (years), mean ± SD	31.7 ± 4.3	29.9 ± 5.4	32.1 ± 3.9	* < 0.001*
Advanced maternal age status, *n* (%)				0.495
No (< 35 years old)	499 (75.7)	93 (78.2)	406 (75.2)	
Yes (≥ 35 years old)	160 (24.3)	26 (21.9)	134 (24.8)	
Parity, *n* (%)				*0.004*
0	551 (83.6)	89 (74.8)	462 (85.6)	
> 1	108 (16.4)	30 (25.2)	78 (14.4)	
Gestational age at delivery (weeks), mean ± SD	39.3 ± 1.1	39.3 ± 1.2	39.3 ± 1.1	0.780
Birthweight (g), mean ± SD	3516.0 ± 456.6	3497.3 ± 409.5	3520.1 ± 466.6	0.623
Delivery method, *n* (%)				* < 0.001*
Spontaneous vaginal	471 (71.5)	66 (55.5)	405 (75.0)	
Vacuum-assisted	146 (22.2)	37 (31.1)	109 (20.2)	
Forceps-assisted	32 (4.9)	14 (11.8)	18 (3.3)	
VBAC	10 (1.5)	2 (1.7)	8 (1.5)	
Laceration type, *n* (%)				0.763
3a	331 (50.2)	64 (53.8)	267 (49.4)	
3b	134 (20.3)	22 (18.5)	112 (20.7)	
3c	139 (21.1)	25 (21.0)	114 (21.0)	
Third degree (unspecified)	5 (0.8)	0 (0)	5 (0.9)	
Fourth degree	50 (7.6)	8 (6.7)	42 (7.8)	
Episiotomy, *n* (%)				* < 0.001*
None	559 (84.8)	77 (64.7)	482 (89.3)	
Medial	58 (8.8)	30 (25.2)	28 (5.2)	
Mediolateral	42 (6.4)	12 (10.1)	30 (5.6)	
Race, *n* (%)				* < 0.001*
White	431 (65.4)	66 (55.5)	365 (67.6)	
Black	55 (8.4)	21 (17.7)	34 (6.3)	
Asian	100 (15.2)	12 (10.1)	88 (16.3)	
Other/unknown	73 (11.1)	20 (16.8)	53 (9.8)	
Ethnicity, *n* (%)				*0.003*
Non-Hispanic	605 (92.4)	102 (85.7)	503 (93.8)	
Hispanic	50 (7.6)	17 (14.3)	33 (6.2)	
Primary language, *n* (%)				0.057
English	622 (94.4)	108 (90.8)	514 (95.2)	
Non-English	37 (5.6)	11 (9.2)	26 (4.8)	
Insurance type, *n* (%)				* < 0.001*
Private/self-pay	557 (84.5)	79 (66.4)	478 (88.5)	
Medicaid/state assistance	102 (15.5)	40 (33.6)	62 (11.5)	
Average Edinburgh Postpartum Depression Scale score, mean ± SD	3.86 ± 3.46	4.98 ± 4.48	3.70 ± 3.26	*0.005*
Postpartum depression screening results, *n* (%)				* < 0.001*
Low (EPDS 0–9)	478 (92.6)	54 (81.8)	424 (94.2)	
High (EPDS ≥ 10)	38 (7.4)	12 (18.2)	26 (5.8)	

*EPDS* Edinburgh Postpartum Depression Scale

Note: Italicized to indicate statistically significant *p*-value at alpha < 0.05

During the time period, 540 out of 659 (81.9%) followed up in PPRC and 119 (18.1%) did not. Patients who attended PPRC were significantly older than those who did not (mean 32.1 ± 3.9 years vs 29.9 ± 5.4, *p* < 0.001) and were more likely to be primiparous (*p* = 0.004; Table 1). Failure to follow up was associated with multiparity (OR 0.50, 95% CI 0.31–0.81), Black race (OR 0.29, 95% CI 0.16–0.54), Hispanic ethnicity (OR 0.39, 95% CI 0.21–0.73), having Medicaid or state insurance (OR 0.26, 95% CI 0.16–0.41), and EPDS score ≥ 10 (OR 0.28, 95% CI 0.13–0.58) on univariable analysis (Table 2). On multivariable analysis, having Medicaid or state medical assistance (aOR 0.43, 95% CI 0.19–0.96) and EPDS score ≥ 10 (aOR 0.30, 95% CI 0.13–0.68) remained associated with failure to follow up in the PPRC (Table 2).

**Table 2:** Factors associated with postpartum recovery clinic (PPRC) attendance

	Univariable OR, OR (95% CI)	Multivariable OR^*^, aOR (95% CI)
Advanced maternal age (age ≥ 35 years old)	1.18 (0.73–1.90)	0.74 (0.39–1.41)
Multiparity	*0.50 (0.31–0.81)*	0.52 (0.27–1.00)
Race		
White	Reference	Reference
Black	*0.29 (0.16–0.54)*	0.48 (0.18–1.24)
Asian	1.33 (0.69–2.56)	2.16 (0.78–5.97)
Other/unknown	*0.48 (0.27–0.85)*	0.72 (0.26–1.95)
Hispanic ethnicity	*0.39 (0.21–0.73)*	0.58 (0.21–1.55)
Non-English speakers	0.50 (0.23–1.04)	1.05 (0.33–3.39)
Medicaid/state assistance insurance	*0.26 (0.16–0.41)*	*0.43 (0.19–0.96)*
High (EPDS ≥ 10)	*0.28 (0.13–0.58)*	*0.30 (0.13–0.68)*

*EPDS* Edinburgh Postpartum Depression Scale, *OR* odds ratio, *aOR* adjusted odds ratio, *CI* confidence interval

^*^Adjusted for multiparity, race, ethnicity, insurance status and high EPDS score

Note: Italicized to indicate statistically significant *p*-value at alpha < 0.05

We also examined factors associated with failure to follow up within the recommended 3-week period (Table 3). Of the patients who followed up, 468 (86.7%) followed up within 3 weeks and 72 (13.3%) after. The mean time to follow-up was 12.7 days (range 1–124 days). On univariable analysis, the only factor associated with failure to follow up within 3 weeks was having Medicaid or state insurance (OR 0.34, 95% CI 0.18–0.64). After controlling for insurance status, patients who were of advanced maternal age (defined as patients who are 35 years or older at time of delivery) were significantly less likely to follow up within 3 weeks (aOR 0.51, CI 95% 0.29–0.89).

**Table 3:** Factors associated with postpartum recovery clinic (PPRC) follow-up within 3 weeks

	Univariable OR (95% CI)	Multivariable aOR^*^ (95% CI)
Advanced maternal age (age ≥ 35 years old)	0.61 (0.36–1.05)	*0.51 (0.29–0.89)*
Multiparity	0.73 (0.38–1.41)	0.85 (0.43–1.67)
Race		
White	Reference	Reference
Black	0.62 (0.24–1.59)	1.07 (0.37–3.08)
Asian	0.84 (0.43–1.68)	1.09 (0.52–2.26)
Other/unknown	0.51 (0.24–1.06)	0.80 (0.34–1.86)
Hispanic ethnicity	0.67 (0.27–1.68)	0.89 (0.34–2.32)
Non-English speakers	0.63 (0.23–1.73)	1.46 (0.46–4.56)
Medicaid/state assistance insurance	*0.34 (0.18–0.64)*	*0.34 (0.18–0.64)*
High (EPDS ≥ 10)	0.92 (0.31–2.77)	1.12 (0.36–3.44)

*EPDS* Edinburgh Postpartum Depression Scale, *OR* odds ratio, *aOR* adjusted odds ratio, *CI* confidence interval

^*^Adjusted for Medicaid/state insurance

Note: Italicized to indicate statistically significant *p*-value at alpha < 0.05

## Discussion

Our study found a high overall attendance rate (81.9%) to a urogynecologist-led postpartum recovery clinic in birthing people who sustained OASIS, with most following up within 3 weeks. Although the high overall attendance rate is favorable, we did identify important disparities in attendance rates, with lower odds for PPRC attendance among patients with Medicaid or state insurance and patients with elevated EPDS scores.

Our high attendance rate contrasts with the relatively low reported general postpartum follow-up rates in most studies; however, there is variation [12]. For example, a study of 4049 people who received prenatal care at a large academic center found that overall, only a third attended a postpartum appointment [17]. However, in a separate study at a Medicaid-based university clinic, follow-up rates were 70% and increased to 88% after the implementation of a patient-centered navigation program (*n* = 474) [18]. A study of 4005 patients seen at an academic center reported overall postpartum attendance rates of over 80% [19]. At a hospital within the same health system as our present study, a study examining postpartum follow-up for patients with pre-eclampsia reported a 6-week follow-up rate of 52% [20]. The higher attendance rate seen in our present study suggests that birthing people with OASIS are motivated to seek short-interval specialist follow-up care if it is available. Our findings are similar to a study comparing patients with and without OASIS (*n* = 2013), which found that those with OASIS were more likely to attend a postpartum visit (86.1% vs 80.0%, *p* < 0.05) [21]. These are reassuring findings and should encourage other institutions who are considering creating a postpartum perineal clinic, especially considering that specialized postpartum pelvic-floor clinics have been described as a sustainable model of care [10].

We found that insurance status was a barrier to follow-up, with patients who have Medicaid or state medical assistance being less likely to follow up and less likely to follow up within the recommended 3 weeks, despite both forms of insurance being accepted at our PPRC. This is similar to a recent study at an urban urogynecology practice comparing characteristics of patients who missed appointments versus those who attended their appointments (*n* = 426) [22]. In addition to demographic characteristics (such as younger age, Black race, Hispanic ethnicity, and being non-English speakers), having Medicaid insurance was a predictor of missing appointments (aOR 2.11, 95% CI 1.04–4.48, *p* = 0.044) [22]. Compared with private insurers, Medicaid insurance programs cover more patients with low socioeconomic status, which makes having Medicaid a strong indicator of financial hardship [17, 23]. A study assessing an initiative aimed at increasing attendance at postpartum visits among Medicaid participants identified barriers to access such as lack of patient information on services and referrals covered, inefficient scheduling processes, and logistical concerns [24]. Scheduling postpartum visits prior to hospital discharge and providing patients with the date and time of their postpartum visit have been shown to improve follow-up rates [25]. This assisted scheduling approach may be particularly helpful for patients seeking specialist care in a postpartum perineal clinic, to help clarify concerns about insurance coverage, and provide early referrals if needed. Logistical barriers to postpartum clinic attendance may include lack of transportation and difficulty finding affordable childcare [24]. For patients at our urban institution, traveling by car to the clinic may be cost prohibitive owing to parking expenses, rideshare fares, or taxi fares. The use of public transportation may be unappealing because of discomfort due to OASIS or difficulty traveling with a newborn if affordable childcare is unavailable. To overcome some of these logistical barriers, especially for lower-income patients who are insured by Medicaid, a potential solution may be to provide vouchers for parking or public transportation. Institutions planning to establish a postpartum perineal clinic should carefully consider its location. For instance, our PPRC clinic is situated within the same facility where many patients received prenatal care, providing them with a familiar and easily accessible setting.

Additionally, we found that patients with an elevated EPDS were less likely to follow up in a PPRC. A prior study by Swenson et al. found that in patients referred to a perineal clinic, 15.6% had a positive EPDS screen (≥ 10) [26], suggesting a high prevalence of depression among postpartum patients with pelvic-floor dysfunction. A recent study comparing patients with and without OASIS found no difference in depression at 1 month and 6 months postpartum; however, this study used a higher EPDS cut-off score to define depression [21]. The authors used a threshold of 13 to define depression, but in our study, we used a threshold of 10 to avoid missing cases of minor or major depression [14, 27]. There are factors unique to having OASIS that may predispose patients to a depressed mood. A qualitative study of people who experienced OASIS found that patients may struggle with the feeling of a “damaged body,” and grappling with issues such as sexual dysfunction and poor self-image [28]. Participants in that study identified having an attentive and helpful provider within the health care system as an important positive factor in their path to recovery [28]. Considering the psychological impacts of OASIS, birthing people with OASIS who are at an elevated risk for depression stand to benefit significantly from attending a PPRC. Again, assistance with scheduling an appointment in PPRC prior to hospital discharge may help to unburden this high-risk group and improve follow-up rates. Patient navigation services have been associated with improved postpartum appointment attendance in patients with antenatal depressive symptoms [29].

On univariable analysis we found that Black race and Hispanic ethnicity were associated with lack of follow-up; however, after controlling for other factors (multiparity, insurance status, and elevated EPDS) there were no differences in follow-up by race/ethnicity. Furthermore, there were no differences in 3-week follow-up by race or ethnicity. This finding contrasts with those of other studies, which have found racial and ethnic disparities both in postpartum visit attendance [20, 24] and in care for PFDs [30]. In a systematic review of patients seeking care for PFDs, younger patients and racial/ethnic minorities had lower care-seeking rates [30]. Mou et al. described a health-equity-based conceptual framework for postpartum PFD care that includes a complex, layered interaction of societal, community, and interpersonal factors [13]. The authors identified limited health care access as a shared risk factor for both PFD care and postpartum care; therefore, specialized postpartum pelvic-floor clinics are poised to improve health equity by increasing access to specialized PFD care [13]. Our findings suggest that routine referral to a postpartum perineal clinic for all patients with OASIS might promote equitable care for patients who self-identify as being from a racial or ethnic minority.

Strengths of this study include a large cohort of over 500 patients seen over a 6-year period. Limitations innate to the retrospective nature of this study include reliance on the completeness of the EMR for demographic and obstetric data. This is particularly relevant for the EPDS scores, which were not routinely collected prior to hospital discharge at our institution until 2019, and information regarding patients’ educational level, which is not collected routinely at our institution. Furthermore, we were limited to using data in our current EMR and did not have sufficient data to compare our cohort with a cohort of patients prior to the establishment of our PPRC, which may have better characterized the impact of having a specialized PPRC on follow-up rates. Additionally, this study was conducted at a single institution, where most of our study population identify as white and non-Hispanic, which may limit generalizability. Finally, although there is clear utility in short-interval follow-up for patients with OASIS in terms of identifying and treating wound complications, evaluation in the initial postpartum period is too soon to identify the long-term complications of OASIS, such as prolapse, persistent anal incontinence, and dyspareunia (as patients are less likely to have attempted intercourse during the early postpartum period). Despite this, evaluation by a urogynecologist in the immediate postpartum period allows patients to be educated about these long-term sequelae, be referred for additional care such as pelvic-floor physical therapy, and understand where to seek care for potential pelvic-floor disorders in the future.

In conclusion, this study found a high overall attendance rate to a urogynecologist-led PPRC in birthing people who sustained OASIS. Factors associated with failure to follow up in this population include an elevated EPDS score and having Medicaid or state medical assistance. Future work should focus on ways to improve attendance in these vulnerable groups by addressing the additional barriers faced by those with Medicaid insurance and mental-health challenges to receiving specialized postpartum care. Health care systems may accomplish this by assisting with scheduling urogynecology appointments for patients who sustained OASIS, and addressing any questions or concerns about insurance coverage during scheduling. Providing patient navigation services and translation services where applicable are additional interventions that promote equitable care and increase access. Educating patients before leaving the birthing facility about the benefits of short-interval, specialized follow-up with a urogynecologist may also encourage improved attendance, particularly for patients with lower health literacy. Thoughtful interventions aimed at improving access and attendance to specialized postpartum care for patients with OASIS have the potential to improve long-term outcomes for this patient population.

## Data Availability

The dataset analyzed for the current study can be made available from the corresponding author upon reasonable request.
